# Editorial: Biomass Resources Utilization

**DOI:** 10.3389/fchem.2022.948565

**Published:** 2022-06-28

**Authors:** Kai Yan

**Affiliations:** Guangdong Provincial Key Laboratory of Environmental Pollution and Remediation Technology, School of Environmental Science and Engineering, Sun Yat-sen University, Guangzhou, China

**Keywords:** pretreatment, bio-oil, value-added chemicals, utilization, catalysis

Biomass utilization is one of the pivotal loops in many biorefinery schemes for the production of value-added chemicals and biofuels (Huber et al., 2006; Alonso et al., 2012; Hu et al., 2021). The effective utilization of biomass resources including the understanding of complex structures and mild fractionation into individual components is a profound challenge for the development of biorefinery (Climent et al., 2010; Hu et al., 2019; Abu-Omar et al., 2020). Challenges are often associated with secondary pollution in the pretreatment as well as the utilization process, whereas harsh reaction conditions, low product selectivity, and poor catalyst stability are also major headaches (Climent et al., 2014; Liu et al., 2021a; Yan et al., 2021). Developing a green and efficient catalytic system to improve conversion efficiency and reduce environmental pollution is crucial (Akhtar and Amin 2011; Alonso et al., 2013; Wang et al., 2019; Yi et al., 2019; Liu et al., 2021b). Moreover, using a biorefinery approach to achieve the integration of the process of biomass conversion with the production of biofuels and chemicals is more practical (Gallezot 2012; Huang et al., 2020; Li et al., 2021; Li et al., 2022a; Li et al., 2022b). This special issue mainly focuses on the pretreatment of biomass, conversion of biomass waste or biomass-based sources or platform chemicals to value-added products and biofuels, highly valued materials in various environmental remediation.

## Biomass Pretreatment

Dong et al. firstly used a two-step pretreatment process to increase the digestibility of Brewer’s spent grains (BSG), high-fiber meal (HFM), and dry distillers grains and solubles (DDGS). BSG, HFM, and DDGS were treated with alkali at 100°C for breaking the ester linkages, followed by an ammonia fiber expansion (AFEX) process. The effect of the alkali amount was investigated, and the use of 4% alkali was favorable to de-esterify biomass. It was of greater importance that the content of carboxamide was similar to the untreated biomass, which was 0.04 mg/g. In addition, the two-step pretreatment process enhanced the glucan conversion. It provided a feasible idea for the treatment of industrial lignocellulosic biomass byproducts.

Tan et al. reviewed various methods (chemical, physical, biological, and combined treatments) for the pretreatment of straw biomass for fermentable sugars production. They evaluated each treatment method in detail, and their advantages and disadvantages were given. Chemical pretreatment was economic but harmful to the environment. Biological pretreatment was environmentally benign but the process was too long. The combined treatments were more efficient than the single method due to synergies. They suggested that the future pretreatments should focus on the following traits: 1) strengthening the basic research on the structure of lignocellulosic biomass at the molecular level, 2) Identifying and analyzing the unknown inhibitory components in the biomass for decreasing the pretreatment cost, 3) using computational tools to optimize the pretreatment process, 4) exploring the novel treatment approaches to improving the conversion of lignocellulosic biomass, 5) investigating the reaction mechanism of emerging methods for the treatment of biomass.

## Bio-Oil Production

Zhao et al. reported that phenol-rich bio-oil was selectively generated by the thermal pyrolysis of corn straw in a homemade microwave. A measure of 46.7 wt% bio-oils was selectively produced and the experimental results confirmed temperature versus heating rate had deciding effects on the selectivity of phenolic oil with the optimal selectivity of 49.4 area%. Photocatalysis was an environmentally friendly and energy-saving biodiesel production method. Huang et al. reviewed the latest progress in photocatalytic biodiesel production from three aspects: transesterification mechanism, photocatalytic type, and modification strategy. At the same time, some problems in the production of biodiesel by the photocatalytic method are highlighted. For example, the mechanism of photocatalytic (trans) esterification had not been systematically studied, and photocatalysts usually contain expensive precious metals. Overall, photocatalytic biodiesel production is still in its infancy, but opens a new door for green biofuel production.

### Value-Added Chemicals

A variety of biomass-derived chemicals (e.g., furfural, 5-hydroxymethyfurfural (HMF), levulinic acid (LA)) can be selectively produced from biomass and further upgraded into highly value-added furfuryl alcohol (FOL), γ-valerolactone (GVL) and 2,5—dimethylfuran (DMF). Gao et al. prepared a bimetallic PtNi/SBA-15 catalyst for the hydrogenation of furfural and HMF by a two-step method. After calcination at 500°C in air, PtNi/SBA-15 was obtained without reduction, achieving 77.9% FOL yield and 81.9% DHMF yield. The electron-rich Pt^δ−^ species on PtNi/SBA-15 not only favored the adsorption and activation of the carbonyls in FF/HMF but also promotes the activation of the hydrogen molecule, which was of great significance for the upgrading and transformation of aldehydes. Hu et al. developed a post-synthetic strategy to synthesize ZrY for the efficient hydrogenation of LA into fine chemical GVL, whereas over 95% yield of GVL was obtained. The parameters of acidity and the metal-support interaction were the deciding factors in the production of GVL.

## Environmental Application

Liu et al. successfully recovered Ni from laterite ore efficiently by H_2_ reduction with sodium thiosulfate (Na_2_S_2_O_3_). They found that the use of 20 wt% Na_2_S_2_O_3_ could efficiently recover Ni from laterite ore, achieving a Ni content of 9.97% and a Ni recovery of 99.24% at 1,100°C. They investigated the effects of reaction conditions (temperature, reduction time, and Na_2_S_2_O_3_) on the nickel laterite reduction, and the mechanism of Na_2_S_2_O_3_ in promoting the reduction of laterite ore by H_2_ was concluded.

In the process of removing antibiotics in wastewater treatment, many factors affect the adsorption efficiency of biochar (Chen et al., 2021; Li et al., 2021). Sun et al. proved that coconut shell biochar after KOH activation and FeCl_3_ magnetization had a strong removal effect on sulfonamide antibiotics (SA). The maximum adsorption capacities for sulfadiazine, sulfamethazine, and sulfamethoxazole were 294.12, 400.00, and 454.55 mg g^−1^, respectively, five to seven times higher than those achieved with raw biochar. They found that the SAs were adsorbed on the modified biochar via hydrogen bonds between SA molecules and -OH/-COOH groups in the biochar. Mechanistic analysis showed that π+-π EDA, CAHB, electrostatic, and Lewis acid-base interactions may be the main reasons for adsorption of SAs on 50MBC-KOH2.5. It provides a new way to utilize biochar for wastewater treatment.
